# Synthesis, Characterization, and Electrochemical Evaluation of Copper Sulfide Nanoparticles and Their Application for Non-Enzymatic Glucose Detection in Blood Samples

**DOI:** 10.3390/nano13030481

**Published:** 2023-01-25

**Authors:** Phumlani Tetyana, Ntsoaki Mphuthi, Abongile Nwabisa Jijana, Nosipho Moloto, Poslet Morgan Shumbula, Amanda Skepu, Lea Sibulelo Vilakazi, Lucky Sikhwivhilu

**Affiliations:** 1 DSI/Mintek Nanotechnology Innovation Centre, Advanced Materials Division, Mintek, Private Bag X3015, Randburg 2125, South Africa; 2 Department of Chemistry, University of Witwatersrand, Private Bag X3, Braamfontein 2050, South Africa; 3 Department of Chemical Sciences, University of Johannesburg, Doornfontein 2028, South Africa; 4 Department of Chemistry, University of Limpopo, Private Bag X1106, Sovenga 0727, South Africa; 5 Next Generation Health, Division 1, CSIR, Meiring Naude Road, Brummeria, Pretoria 0001, South Africa; 6 Department of Chemistry, Faculty of Science, Engineering and Agriculture, University of Venda, Private Bag X5050, Thohoyandou 0950, South Africa

**Keywords:** copper sulfide, non-enzymatic, glucose sensor, nanoparticles, spiked blood samples

## Abstract

Glutathione-capped copper sulfide (Cu_x_S_y_) nanoparticles with two different average sizes were successfully achieved by using a simple reduction process that involves only changing the reaction temperature. Temperature-induced changes in the size of Cu_x_S_y_ nanoparticles resulted in particles with different optical, morphological, and electrochemical properties. The dependence of electrochemical sensing properties on the sizes of Cu_x_S_y_ nanoparticles was studied by using voltammetric and amperometric techniques. The spherical Cu_x_S_y_ nanoparticles with the average particle size of 25 ± 0.6 nm were found to be highly conductive as compared to Cu_x_S_y_ nanoparticles with the average particle size of 4.5 ± 0.2 nm. The spherical Cu_x_S_y_ nanoparticles exhibited a low bandgap energy (E_g_) of 1.87 eV, resulting in superior electrochemical properties and improved electron transfer during glucose detection. The sensor showed a very good electrocatalytic activity toward glucose molecules in the presence of interference species such as uric acid (UA), ascorbic acid (AA), fructose, sodium chloride, and sucrose. These species are often present in low concentrations in the blood. The sensor demonstrated an excellent dynamic linear range between 0.2 to 16 mM, detection limit of 0.2 mM, and sensitivity of 0.013 mA/mM. The applicability of the developed sensor for real field determination of glucose was demonstrated by use of spiked blood samples, which confirmed that the developed sensor had great potential for real analysis of blood glucose levels.

## 1. Introduction

Diabetes remains one of the most common chronic diseases, accounting for more than 1.5 million deaths each year globally [1]. Diabetes mellitus is a complex global public health crisis that is caused by uncontrolled blood glucose levels and has no known cure. Diabetes affects around 463 million people worldwide and is projected to rise to 578 million by 2030 and to 700 million by 2045, with Type 2 diabetes accounting for up to 90% of the total. The International Diabetes Federation (IDF) reported that approximately 4.58 million people in South Africa have diabetes, constituting about 12.8% of its adult population [2]. Therefore, the availability of a rapid, simple, cheap, disposable, easy-to-use whole-blood assay testing platform for clinical diagnosis, especially in developing countries and resource-limited and remote regions, would greatly benefit point-of-care or public health applications. However, in these new platforms, performance parameters such as stability, selectivity, sensitivity, detection limits, etc., must be balanced with the need for speed, cost, and simplicity [3,4].

In conventional electrochemical biosensors for glucose, glucose oxidase (GOx) immobilized on the electrode surface acts as a catalyst for the oxidation of glucose in the presence of oxygen molecules. The glucose molecule is oxidized at the electrode’s interface, resulting in the formation of gluconolactone and hydrogen peroxide [5]. The main limitations to enzyme-based biosensors is the enzyme’s activity and stability, which is often affected by changes in the medium’s pH, as well as changes in the temperature and humidity of the electrode surface [6,7]. To address these shortcomings, research has progressed to the use of nanomaterial-based catalysts for development of non-enzymatic glucose sensors. These sensors rely on the direct electrocatalytic oxidation of glucose by nanomaterials and nanostructures immobilized on the electrode surface. This is expected to provide convenience and a benefit by eliminating the difficulty of maintaining stability during enzyme preservation [8,9].

Metal nanoparticles, metal oxide nanostructures, and metal-based nanocomposites of Au, Fe, Ni, Pt, Cu, and Zn, to name a few, have caught the interest of researchers due to their ability to directly oxidize glucose molecules effectively [5,6]. Metal-based nanomaterials as sensing electrodes for glucose have several advantages over glucose enzymatic biosensors, such as cost effectiveness, faster electron transfer rates (due to a thinner layer of catalyst mobilized on the electrode surface), simplicity, stability, and high sensitivity [10,11]. These nanomaterials are widely used in the fabrication of electrodes due to their catalytic and conductive properties, which have been shown to improve signal amplification [6,10]. Furthermore, they have a high surface-to-volume ratio, which improves electrocatalytic activity and sensing response through the rapid movement of analytes on the surface of a nanomaterial modified electrode [10,12]. According to Rassaei et al. [13], using nanomaterials to modify electrode surfaces improves electron transfer kinetics, reduces overpotential, increases electroactive surface area, and makes redox reactions kinetically feasible.

Cu-based nanomaterials are gaining popularity over other metal-based nanomaterials such as Au, Pd, Ni, and Pt due to their abundance, low cost, and inherent optical and electrical properties [7,14]. They have exhibited impressive catalytic capability in detecting a range of glucose concentrations due to a good peroxidase imitative performance [6,15,16,17]. Additionally, they have demonstrated the ability to catalyze the breakdown of hydrogen peroxide, making them ideal for the development of biosensing platforms [15]. Among Cu-based nanomaterials, copper sulfide (Cu_x_S_y_) nanoparticles have been identified as promising candidates in electrochemical biosensing applications due to their easy synthesis and size- and shape-dependent tunable bandgap [18]. Cu_x_S_y_ is a p-type semiconductor that exists in various stoichiometric ratios. It is known to exhibit a stoichiometry-dependent bandgap that can be tuned from 1.2 to 2.5 eV [19,20]. In comparison to other potential stoichiometric compositions of Cu_x_S_y_, the structure of covellite (CuS) is reported to be sulfur-rich, with a higher electrical conductivity and stability of up to 500 °C [20]. CuS nanostructures have been shown to have a facile charge transfer mechanism and electrical conduction in electrochemical-sensor applications [21,22]. Furthermore, they have proven exceptional catalytic activity and the redox nature of Cu^2+/^Cu^3+^ as a useful mediator of electron transfer during the electrocatalytic oxidation of glucose [22,23]. In this context, CuS nanostructures have proven to be a potential candidate for the fabrication of glucose sensors without the use of enzymes. This nanomaterial’s properties can be tuned by adjusting its structure, morphology, stoichiometric composition, and valance state [16,19,21,24]. Liu et al. [25] compared the electrochemical properties of CuS nanotubes with different diameters for glucose detection; the results showed that the morphology of the nanostructure plays a significant role in the electrocatalytic oxidation of glucose.

Recent research has focused on CuS with a combination of binders [22], dopants [26], and other nanomaterials [21,27] as support catalysts to improve the electrochemical performance of CuS toward glucose detection. Although doping, nanocomposite, and functionalized nanomaterials have been proven to enhance the conductivity of the electrode surface, the binders and dopants tend to increase the thickness of the electrode films. This significantly leads to the proneness of the materials to surface saturation or fouling. The significance of these hybrid catalysts is also lessened by drawbacks such as complicated synthesis processes, lack of reproducibility, low yield, and poorly understood mechanisms of interaction with biological molecules.

More studies are required to optimize the composition, structure, and distinct properties of the different components in these hybrid catalysts. Additionally, sensors that do not contain a complex mixture of catalysts can be advantageous in terms of biological compatibility, structural simplicity, quality control for large-scale production, and reduced production costs. We suggest that by studying and optimizing the structural morphology, shape, and size of CuS nanoparticles, one can tune conductivity and catalytic activity to achieve greater sensing capabilities.

In this study, two different average sizes of spherical glutathione capped Cu_x_S_y_ nanoparticles were synthesized and explored as electrode materials without support catalysts for the real-time detection glucose. The effects of size and bandgap on electrochemical properties of spherical Cu_x_S_y_ nanoparticles were investigated by using cyclic voltammetry. Glutathione played a significance role in enhancing the stability of CuS nanoparticles on the electrode surface due to presence of both NH_2_ and COOH functional groups, which electrostatically and efficiently interacted with the surface of screen-printed carbon electrode. The presence of both NH_2_ and COOH functional groups from glutathione, in turn, mediated the stability of the sensor material at physiological pH conditions.

## 2. Experimental Procedure

### 2.1. Chemicals

Copper chloride (CuCl_2_·2H_2_O), sodium diethyldithiocarbamate (SDEDTC), glutathione (GSH), sodium hydroxide (NaOH), uric acid (UA), ascorbic acid (AA), fructose, sucrose, sodium chloride (NaCl), phosphate-buffered saline tablets (PBS), potassium ferricyanide, D- (+) -glucose powder, and ethanol were purchased from Sigma-Aldrich (Johannesburg, South Africa) and used without prior modification.

### 2.2. Methodologies

#### 2.2.1. Synthesis of Cu_x_S_y_ Nanoparticles

Copper sulfide nanoparticles were synthesized by using copper chloride and sodium diethyldithiocarbamate as copper and sulfur sources, respectively, with deionized water (dH_2_O) as a solvent. GSH was used as a capping ligand to prevent particles from aggregation. In a typical synthesis method, a solution of GSH (2.4 µmols) was introduced into a three-necked round-bottom flask and stirred at room temperature, followed by a drop-wise addition of a solution of CuCl_2_·2H_2_O (0.6 µmols). After addition of CuCl_2_·2H_2_O solution, the reaction was stirred further for a minute, and then a solution of SDDTC (1.2 µmols) was added dropwise into the flask, and the pH of the reaction was adjusted to 9.0 by a dropwise addition of a 1 M solution of NaOH. The reaction temperature was then adjusted to 50 °C and refluxed for 60 min under nitrogen to promote nanoparticle growth. Nanoparticles were separated via centrifugation, and precipitates were washed three times with ethanol and dispersed in dH_2_O. In a separate reaction, the same protocol was followed, but the reaction was refluxed at 95 °C for 60 min.

#### 2.2.2. Electrode Modification and Electrochemical Measurements

Separate suspensions of as-synthesized Cu_x_S_y_ nanoparticles were prepared by dissolving 5 mg of powdered nanoparticles in 1.5 mL deionized water and sonicated for 30 min. Then 5 µL drops of the prepared suspensions were dropped on the surface of the screen-printed carbon electrode (SPCE) and allowed to dry at room temperature.

#### 2.2.3. Preparation of Real Blood Samples

The samples were collected by using the Venipuncture procedure. Blood samples were immediately transferred to Vacutainer tubes after extraction. Five 0.5 mL raw blood samples were transferred into different 2 mL Eppendorf tubes. The blood samples were then spiked with 1 mL of 0.5, 1, 2, 3, and 4 mM of standard glucose concentrations. All prepared blood samples were stored in designated tubes when not in use.

#### 2.2.4. Characterization Techniques

A Thermo Scientific Multiskan GO UV/Vis Spectrophotometer was used to measure the absorption properties of the reaction products. Data were recorded on samples placed in plastic cuvettes with 1 cm path lengths, using dH_2_O as a reference solvent. Photoluminescence was measured using Perkin Elmer LS55 photoluminescence spectra with a xenon lamp (150 W), in quartz cuvettes (1 cm path length), with dH_2_O as a reference solvent. An AJEOL JEM-2100 high-resolution transmission electron microscope (HRTEM) operated at 200 kV was used to image the reaction products. HRTEM samples were prepared by drop-casting diluted samples onto carbon coated 400 mesh nickel grids. Fourier-Transform Infrared (FTIR) spectroscopy was performed by using an Attenuated Total Reflection (ATR)–Fourier Transform Infrared Spectroscope (Thermo Nicolet 5700 FTIR). X-ray diffraction (XRD) measurements were performed on powdered samples by using a Bruker X-ray Diffractometer (D8 Advance, Cobalt X-ray source, Lynx-eye XE detector), carried out at the 2-theta (2θ) level on a D8 diffractometer. All electrochemical experiments were carried out by using a computer-controlled Autolab Potentiostat/Galvanostat PGSTAT 302 N (Eco Chemie, Utrecht, The Netherlands) driven by NOVA 2.0 data-processing software. The electrochemical data were collected by using in-house screen-printed carbon electrodes. Electrochemical impedance spectroscopy (EIS) experiments were recorded in the frequency range between 100 kHz to 0.1 Hz with an amplitude 0.05 V rms sinusoidal modulation.

## 3. Results and Discussion

### 3.1. Morphological Characterization

**Figure 1:**
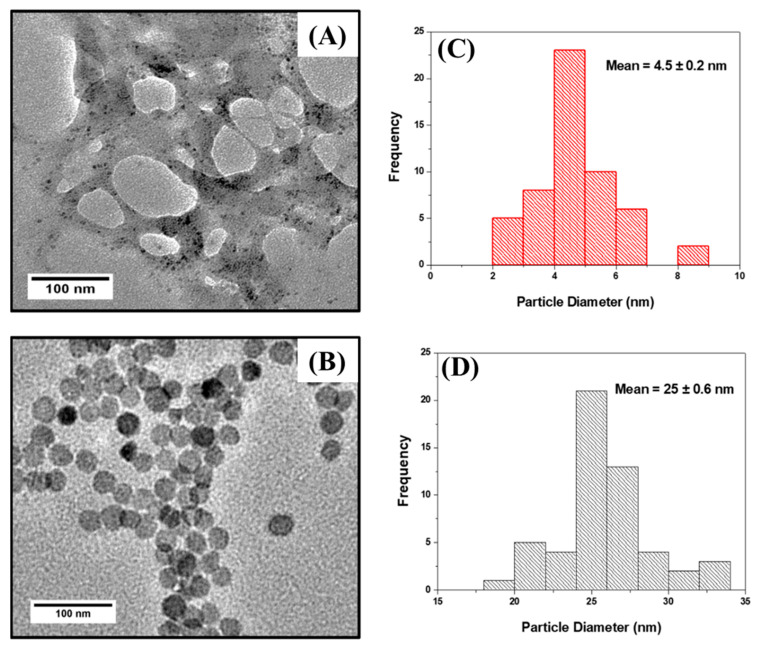
TEM images and particle size distribution of GSH capped (**A**,**C**) CuS-1 and (**B**,**D**) CuS-2.

The temperature and pH are crucial parameters that influence the morphology and subsequent properties of nanoparticles. An increase in the temperature or pH will have an effect on the size and shape of nanoparticles, ultimately influencing the properties of a particular nanomaterial. For instance, high temperatures favor the formation of larger nanoparticles, a phenomenon attributed to Ostwald ripening [28]. Figure 1 shows HR-TEM micrographs of Cu_x_S_y_ nanoparticles synthesized at 50 °C (CuS-1) (Figure 1A) and 95 °C (CuS-2) (Figure 1B), with their respective particle-size distribution histograms (Figure 1C,D). Spherical nanoparticles with an average diameter of 4.5 ± 0.2 nm were formed when the reaction temperature was maintained at 50 °C. The particles appear to be enclosed within a polymer-like material, which is attributed to excess capping ligand (GSH) that has not been used up during synthesis or was completely washed off during purification process; this can be clearly seen in Supplementary Figure S1, at the 50 nm magnification scale. When the reaction temperature was increased from 50 to 95 °C, well-dispersed spherical nanoparticles with an average diameter of 25 ± 0.6 nm were formed. The growth rate of nanoparticles is directly proportional to the reaction temperature. Thus, an increase in the synthesis temperature increases the particle growth rate, which results in the formation of larger particles [29,30].

### 3.2. Optical Characterization

**Figure 2:**
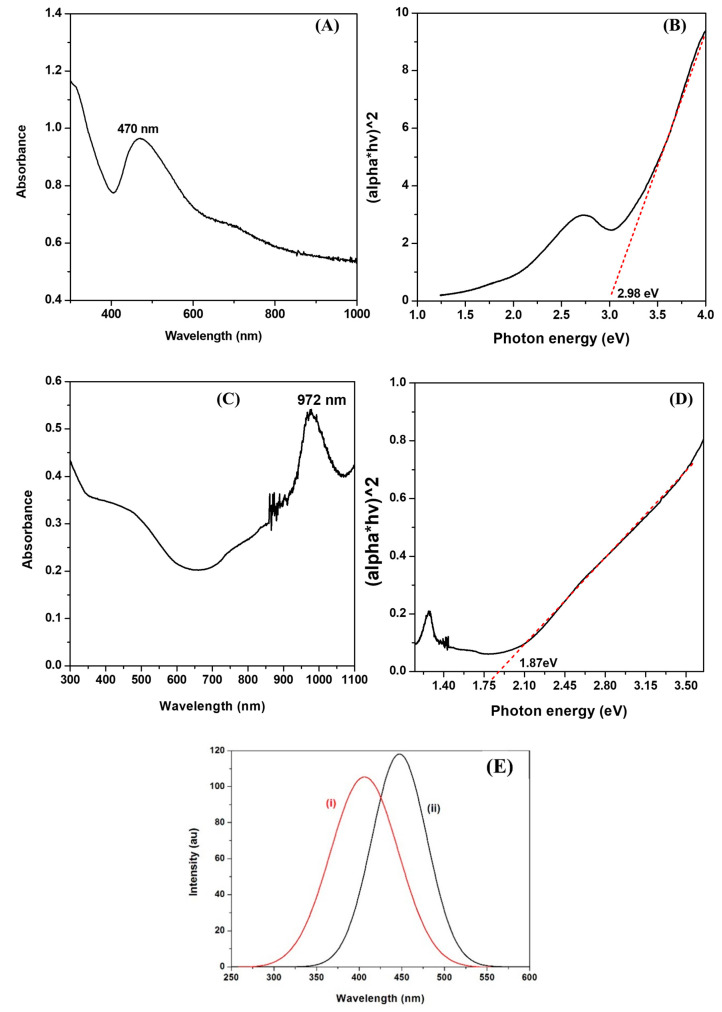
Absorption spectra and Tauc Plots of GSH-capped CuS-1 (**A**,**B**) and CuS-2 (**C**,**D**), respectively. (**E**) Photoluminescence spectra of (i) CuS-1 and (ii) CuS-2.

Figure 2 shows the optical properties of as-synthesized copper sulfide nanoparticles capped with GSH. The absorption spectrum (Figure 2A) of GSH-capped CuS-1 nanoparticles displayed a maximum excitonic peak in the visible region at approximately 470 nm, with a bandgap energy of 2.98 eV (Figure 2B). An emergence of a slightly broad peak in the near-infrared region (NIR), appearing at approximately 700 nm, was also observed. It is noticeable that both peaks are blue-shifted when compared to bulk CuS material, which has a band edge at 1022 nm.

The shift to a lower wavelength is attributed to the quantum confinement of charge carriers in the nanoparticles [31,32,33]. The appearance of two different peaks in the same material is indicative of the presence of both the chalcocite (Cu_2_S) and covellite (CuS) phases. Cu_2_S is known to have an excitonic peak that appears in the visible region (around 470 nm), while CuS is known to appear in the NIR region (around 700 nm) [34]. The absorption spectrum of CuS-2 nanoparticles (Figure 2C), with a bandgap of 1.87 eV (Figure 2D), shows a peak in the NIR region at approximately 972 nm, which can be attributed to an electron-acceptor state in the bandgap [35]. The difference in the absorption spectra of copper sulfide nanoparticles synthesized at 50 and 95 °C signifies a potential change in the properties of the nanoparticles, which is usually due to a change in either size or shape of the nanoparticles as a result of an increase in the growth temperature [36,37,38].

The PL spectra of the as-synthesized Cu_x_S_y_ nanoparticles (λ_exc_ = 350 nm), as shown in Figure 2E, display maximum peaks at 400 nm and 450 nm for particles with an average size of 4.5 nm ((i) CuS-1) and 25 nm ((ii) CuS-2), respectively. These are blue-shifted from their corresponding absorption spectra, a phenomenon that has been reported previously [39]. All emission spectra display single smooth peaks, which indicate that the surfaces of the nanoparticles are well passivated by the capping ligand [40,41]. The photoluminescence (PL) properties of semiconductor nanoparticles are mainly dependent upon their surface states, size distributions, and surface passivation by the capping agent [42].

### 3.3. Structural Characterization

**Figure 3:**
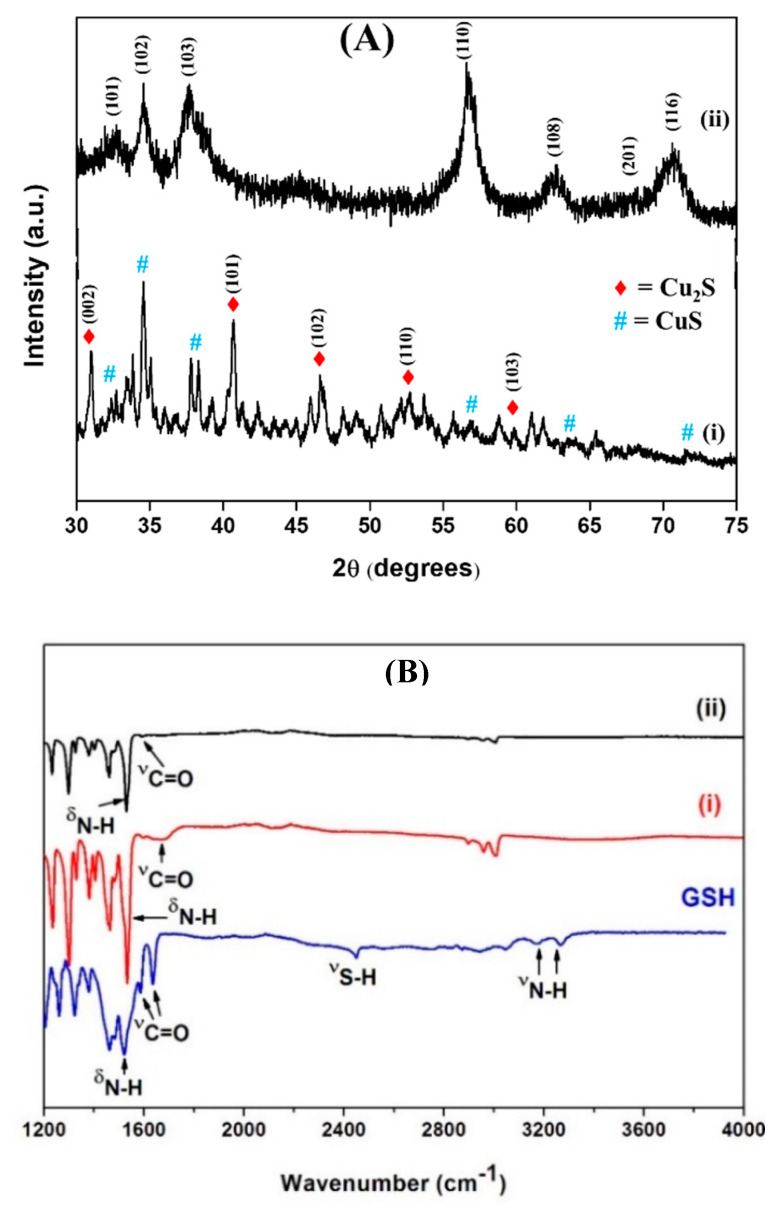
(**A**) XRD analysis of GSH-capped (i) CuS-1 and (ii) CuS-2. (**B**) FTIR spectra of GSH-capped (i) CuS-1 and (ii) CuS-2.

The structural properties of as-synthesized nanoparticles were investigated by using X-ray diffractometer and FTIR spectrometer. The crystallinity and phase composition of as-synthesized Cu_x_S_y_ nanoparticles were examined by using the X-ray diffraction technique (Figure 3A). For CuS-1 nanoparticles (Figure 3A(i)), an emergence of peaks that correspond to various crystal phases, such as the chalcocite (Cu_2_S) and covellite (CuS) phases, were observed. Furthermore, an emergence of small peaks that are attributed to orthorhombic sulfur were identified. This signifies that the reaction is incomplete and there are still traces of the starting material that have not been used up due to the low-reaction temperature used. Low-reaction temperatures decrease reaction kinetics and further slowdown reaction dynamics, resulting in an incomplete reaction [43]. However, a transition to a pure phase of covellite Cu_x_S_y_ was observed for CuS-2 (Figure 3A(ii)). Increasing the reaction temperature facilitated a change in the stoichiometry of the nanoparticles. The diffraction peaks were indexed to crystal planes (101), (102), (103), (110), (108), (201), and (116) of covellite CuS (JCPDS, card number 4-784) [34,44,45]. No traces of impurities were observed in this reaction temperature, and the peaks in the spectra have become narrower, signifying the formation of bigger and crystalline nanoparticles [46,47].

The interaction between glutathione and Cu_x_S_y_ nanoparticles was investigated by using FTIR. Figure 3B shows the FTIR spectra of pure GSH and GSH-capped Cu_x_S_y_ nanoparticles. For GSH only, peaks appearing at 3129 and 3024, 2522, 1718 and 1608, and 1547 cm^−1^ are attributed to the presence of the N-H (NH_3_^+^) stretching band ν(N-H), S-H stretching band ν(S-H), the C=O stretching band of the carboxylic group ν(C=O), and the N-H deformation of the amide bond δ(N-H), respectively. The spectra of GSH-capped Cu_x_S_y_ nanoparticles show similar peaks, but the N-H and S-H stretching bands have disappeared, indicating that GSH interacts with the CuS nanoparticles via the S-H and N-H functional groups, and the peak of ν(C=O) becomes relatively broad. The C=O peak appears on all spectra but has shifted from position 1705 cm^−1^ in the spectra of GSH to a slightly lower frequency in the spectra of Cu_x_S_y_-capped nanoparticles [48,49].

### 3.4. Electrochemical Characterization of the Cu_x_S_y_-Modified Screen-Printed Carbon Electrodes

**Figure 4:**
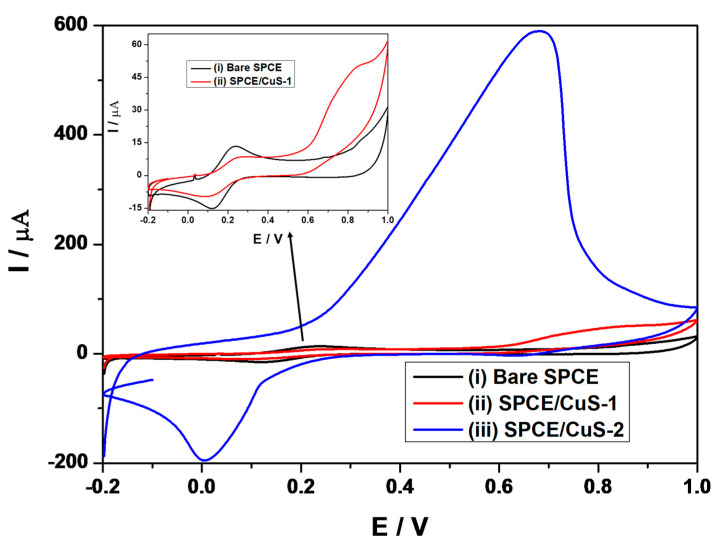
Cyclic voltammetry plots of (i) bare and (ii) CuS-1-, and (iii) CuS-2-modified SPCE in 1 mM K_3_[Fe(CN)_6_]/K4[Fe(CN)_6_] redox probe containing 1 M of KCl, at a potential window between −0.2 and 1 V, at a fixed scan rate of 0.05 Vs^−1^. Insert: cyclic voltammogram highlighting the SPCE/CuS-1 and the bare SPCE.

Cyclic voltammetry measurements of bare and Cu_x_S_y_-modified SPCE were carried out in a 1 mM K_3_[Fe(CN)_6_]/K4[Fe(CN)_6_] redox probe containing 1 M of KCl, at a potential window between −0.2 and 1 V, at a fixed scan rate of 0.05 Vs^−1^. The Cu_x_S_y_ nanoparticles prepared at different temperatures were assigned to CuS-1 and CuS-2, indicating nanoparticles prepared at 50 °C and 95 °C, respectively, as shown in Figure 4. Cyclic voltammograms of the (i) bare SPCE and (ii) SPCE/CuS-1 and (iii) SPCE/CuS-2 showed typical redox peaks corresponding to the [Fe(CN)_6_]^3−^/[Fe(CN)_6_]^4−^ probe (i.e., reversible Fe^3+^/Fe^2+^ reduction and oxidation processes) at potential differences ΔE_p_(s) of 116 mV, 147 mV, and 674 mV, respectively. SPCE modified with CuS-2 showed a broad enhanced current peak at 0.682 V, which resulted from the presence of CuS nanomaterials on the surface of the SPCE. A similar peak was observed at 0.838 V for SPCE/CuS-1; these peaks are attributed to electrochemical oxidation of different crystal configuration and sizes of Cu_x_S_y_ nanomaterials present on the surface of SPCE. SPCE/CuS-2 further showed a high diffusion coefficient of 1.914 × 10^−14^ cm^2^s^−1^, as compared to 3.736 × 10^−18^ cm^2^s^−1^ and 9.698 × 10^−18^ cm^2^s^−1^ for SPCE/CuS-1 and bare SPCE, respectively. The diffusion coefficient (S1) [50] was calculated to determine the rate at which electrons diffuse from the electrolyte solution to the electrode surface, and the results indicated that the CuS-1 conductive film allows fast electron transfer of redox species from the bulk solution to the electrode’s surface.

The presence of negatively charged sulfur atoms has been shown to improve the electrochemical properties of the materials, which, in this case, are a result of the contribution of sulfur atoms from the CuS nanoparticles and the capping ligand [51]. Furthermore, the difference in electrocatalytic behavior between CuS-2 and CuS-1 is due to the quantum size effect or particle size difference [52,53]. When a particle’s size approaches the Bohr radius, its optical and electrical properties become dependent on its physical dimensions [52,53]. The quantum size effect is most prominent for semiconductors’ nanoparticles, where the bandgap increases as the particle size decreases, indicating that the energy difference between the valence band and the conduction band can be altered as the particle size changes. As a result, the large bandgap of CuS-1 reduced its electrical conductivity. Furthermore, the poor electrical conductivity of CuS-1 might also be due to surface defects. Amelia et al. reported that small particles/quantum dots are prone to surface defects when conducting electrochemical experiments [53]. The improved electrochemical properties of CuS-2 are attributed to a small energy difference between the valence and conduction bands, which made electrons more easily excited into the conduction band and serve as charge carriers to conduct electricity [54]. As a result, conductive pathways for electrons and electrolyte ions were formed, increasing the active surface area, which, in turn, influenced the electrochemical response of CuS-2.

**Figure 5:**
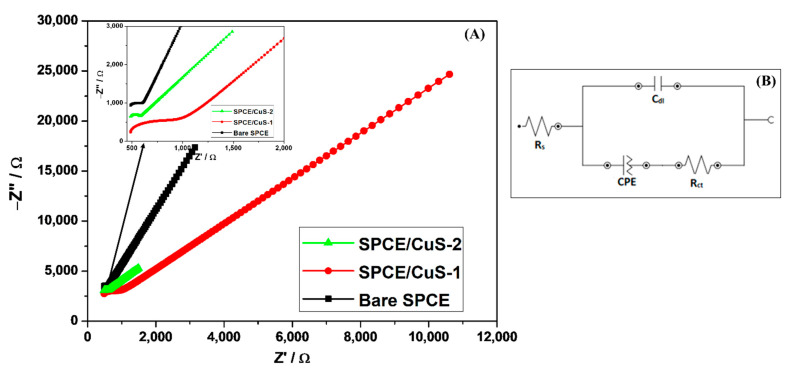
(**A**) Nyquist plots of (i) bare SPCE and (ii) SPCE/CuS-1 and (iii) SPCE/CuS-2 (insert: Nyquist plot highlighting the bare and Cu_x_S_y_-modified SPCE) and (**B**) Randles equivalent circuit in 1 mM K_3_[Fe(CN)_6_]/K_4_[Fe(CN)_6_] redox probe containing 1 M of KCl.

To study the electrochemical kinetics of the Cu_x_S_y_ nanomaterials at the electrode/electrolyte interface, electrochemical impedance spectroscopy (EIS) studies were performed. EIS was recorded over the frequency range from 100 kHz to 0.1 Hz and 0.05 Vrms sinusoidal modulation, using the 1 mM [Fe(CN)_6_]^3−^/[Fe(CN)_6_]^4−^ redox probe. The Nyquist plots and the Randles equivalent circuit of (i) bare SPCE, (ii) SPCE/CuS-1, and (iii) SPCE/CuS-2 are illustrated in Figure 5. The fitted equivalent circuit module parameters are also illustrated in Table 1.

In the Nyquist plot shown in Figure 5A, SPCE/CuS-1 showed the largest semicircle, followed by bare SPCE and then SPCE/CuS-2. The Randles circuit (Figure 5B) showed characteristic elements; solution resistance (R_s_), charge transfer resistance (R_ct_), constant phase element (CPE), and double-layer capacitance (C_dl_). The diameter of the semicircle represents the charge-transfer resistance for the SPCE/electrolyte interface, and the liner part at lower frequencies represents the diffusion process [55]. The charge transfer kinetics occurring at different electrodes’ interfaces were monitored by the distinguished charge-transfer-resistance magnitudes shown in Table 1. The magnitude of R_ct_ differed significantly between electrodes based on the data obtained. The CuS-1-modified electrode showed the highest R_ct_ value (536 Ω) compared to the bare SPCE (201 Ω) and CuS-2 (93.8 Ω) interface in the presence of the [Fe(CN)_6_]^3−^/[Fe(CN)_6_]^4−^ redox probe. The high R_ct_ of SPCE/CuS-1 indicates that it has a low conductivity, whereas the CuS-2 film promotes high electron transfer and improves the conductivity of the electrode. This was consistent with the results obtained from CV studies. Slow electrode kinetics for CuS-1 indicate that a redox charge transfer reaction is primarily controlled by mass transport. In addition, the R_s_ value of SPCE/CuS-2 is lower than that of SPCE/CuS-1, indicating an increased electrocatalytic activity of [Fe(CN)_6_]^3−^/[Fe(CN)_6_]^4−^ redox process on electrode’s surface [56,57].

The electrochemical surface properties and the exchange current rates of the Cu_x_S_y_-modified SPCE interfaces were evaluated with respect to the charge transfer resistance of the unmodified SPCE. The surface coverage (θ) of SPCE/CuS-2 and SPCE/CuS-1 were calculated by using Equation S2 [58]. The SPCE/CuS-2 and SPCE/CuS-1 had surface coverage (θ) of 1.14 and 0.63, respectively. An enhanced electrochemical surface coverage is expected for the SPCE/CuS-2 due to larger-sized nanoparticles compared to the SPCE/CuS-1 that possessed nanoparticles with an average diameter of less than 10 nm. Furthermore, CuS-2 nanostructures were monodispersed and spherical in shape, indicating a high surface concentration of the electroactive species and thus improved electro-catalytic behavior [51,59]. This phenomenon also explains the capacitive behavior of modified electrodes, which is proportional to the surface area of the electrode [60,61]. SPCE/CuS-2 had a relatively high capacitance, which indicates the conductive nature of CuS-2-modified electrode. The CPE replaces the ideal capacitor whenever the electrodes are characterized by rough surfaces (Supplementary Figure S2) [62]. Exchange current densities of 4.79 × 10^−5^ A/cm^2^ and 2.74 × 10^−4^ A/cm^2^ for the SPCE/CuS-1- and SPCE/CuS-2-modified electrode surfaces, respectively, were evaluated. The exchange current densities are dependent on many factors, such as the electrode surface composition, its morphology, the electron transfer reactions occurring at the interface, and the nature of the adsorbed species on the electrode’s surface. The exchange current density describes the equilibrium electron transfer rate of the oxidized and reduced species. The exchange current density parameters were evaluated by using the Butler–Volmer Equation (S3) [62,63,64].

### 3.5. Scan Rate Studies of CuS-2-Modified Electrodes

**Figure 6:**
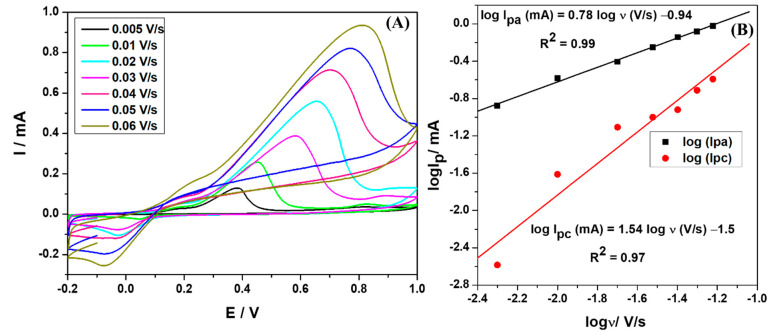
(**A**) Cyclic voltammograms of the SPCE/CuS-2 at different scan rates (0.005 to 0.06 V/s) in 0.01 M phosphate buffer solution of pH 7.4 containing 3 mM of glucose. (**B**) Dependence of the logarithm of redox currents with the logarithm of scan rates.

The CuS-2 nanoparticles were used for the detection of glucose due to their remarkable electrochemical behavior compared to CuS-1 nanoparticles, as established in the previous sections. To study the redox properties of the preferred modified electrode surface, the dependence of the electrochemical activity to the scan rates was conducted by using SPCE/CuS-2-modified electrodes. Figure 6A shows cyclic voltammograms of the CuS-2 recorded at a potential window between −0.2 and 1.0 V; scan rates were varied from 0.005 to 0.06 V/s, and the electrolyte solution consisted of 3 mM glucose in 0.01 M phosphate buffer solution with a pH of 7.4.

As shown in Figure 6A, cathodic and anodic peak currents increased with the increasing scan rates from 0.005 to 0.06 V/s, shifts in both the reduction and oxidation potentials were also observed. The cathodic currents shifted toward greater negative potentials at high scan rates, while the anodic currents shifted to much more positive potential values. These cathodic and anodic potential shifts often result in increased potential differences, as this behavior is attributed to the heterogeneous electron transfer kinetics occurring at the electrode surface, indicating a quasi-reversible redox system. In addition, this confirms the electrocatalytic oxidation of adsorbed glucose molecules onto the surface of CuS-2 nanoparticles mobilized on the electrode interface [65].

The relationship between the logarithm of redox peak currents with the logarithm of the scan rates gave good linear relationships corresponding to the linear equations, Iog *I_pa_* (mA) = 0.78 log *v* (V/s) −0.94 (R^2^ = 0.99) and Iog *I_pc_* (mA) = 1.54 log *v* (V/s) −1.5 (R^2^ = 0.97), for the anodic and cathodic processes, respectively (Figure 6B). The plots of log *I_p_* versus the log v for the anodic and cathodic processes gave slopes of 0.78 and 1.54, respectively, which are ideally close to 1; this indicates that both the reduction and oxidation processes occurred at very thin layers and are dominantly controlled by adsorption [66]. This was further supported by the high Tafel value (*b*) of 806.26 mV dec^−1^ obtained from the Tafel plot of anodic peak potential (E_p_) versus log v (Supplementary Figure S3). The Tafel slope was calculated by using the Tafel equation (S4). The obtained Tafel value is greater than the theoretical value of 118 mV dec^−1^, thus indicating the adsorption of reactants or intermediates on the electrode surfaces and/or reactions occurring within a porous electrode structure [67,68].

### 3.6. Reproducibility of the CuS-2-Modified Electrodes

**Figure 7:**
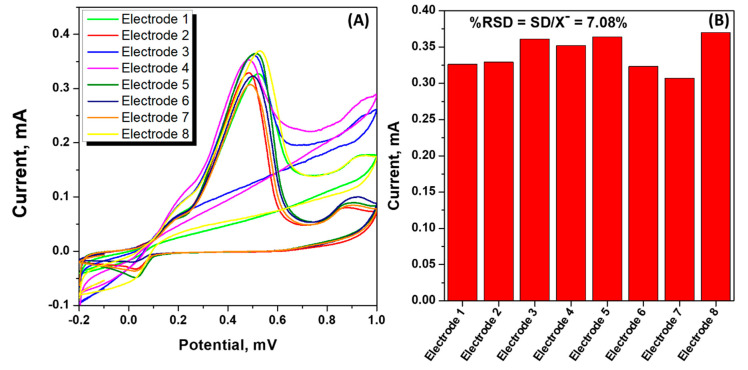
(**A**) Cyclic voltammograms and (**B**) histogram representation of the current responses of eight different SPCE/CuS-2 in 0.01 M phosphate buffer solution containing 3 mM glucose.

Reproducibility plays a significant role during prototyping and development of sensors and biosensors, as it describes the closeness of the obtained data relative to recurring measurements under the equivalent experimental conditions. Eight different electrodes were prepared by the drop-casting CuS-2 onto the working electrode surfaces of the SPCE(s), as described in the preceding sections. CV was used to demonstrate reproducibility studies, and the CV plots were recorded at a potential window between −0.2 and 1.0 V in the presence of 3 mM glucose suspended in the phosphate buffer solution, as highlighted in Figure 7A; a scan rate of 0.03 V/s was used. Figure 7B illustrate the histogram representation of eight different CuS-2-modified electrodes in the presence of 3 mM glucose under the same experimental conditions. All the CuS-2-modified electrodes exhibited well-pronounced oxidation peaks at 0.45 V and 0.56 V. This is attributed to both the oxidation of Cu^+^/Cu^2+^ and glucose molecules [69]; a relatively low standard deviation (RSD) of 7.08% was evaluated. This suggested that the CuS-2-modified SPCE(s)-based sensors for the detection of glucose were highly reproducible and demonstrated good repeatability.

### 3.7. Amperometric Detection of Glucose at CuS-2-Modified Electrodes

**Figure 8:**
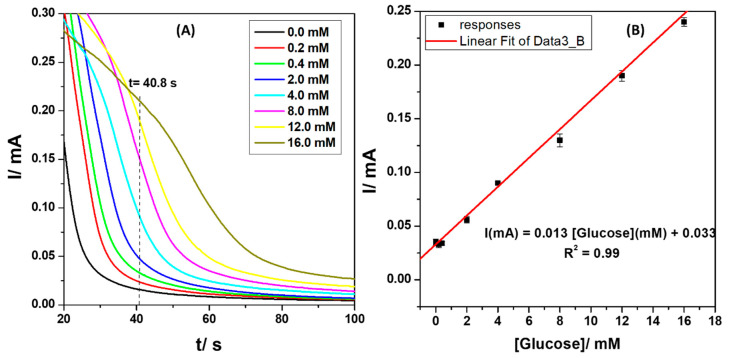
(**A**) Chronoamperometry responses of CuS-2-modified SPCE at different concentrations of glucose (0.2 to 16 mM) in 0.01 M phosphate buffer solution and (**B**) the corresponding calibration curve.

The amperometric studies were carried out to investigate the effect of concentration of glucose on the developed sensor at a fixed potential of 0.65 V and to determine the detection limit (DL) of the sensor toward glucose. Chronoamperometry responses (Figure 8A) were recorded for 100 s in an electrolyte solution containing different concentrations of glucose ranging from 0.2 to 16 mM, prepared in 0.01 M phosphate buffer solution with a pH of 7.4, using different Cu-modified SPCE for each measurement. Figure 8B represents the calibration curve of the modified electrode toward glucose detection, with an electrochemical response time (t) of 40.8 s. The results showed that the current generated was simultaneously increasing with glucose concentrations from 0.2 to 16 mM. This indicates that the sensor demonstrated excellent electrocatalytic behavior and high efficiency toward the electro-oxidation of glucose. A very good linear response was established with a linear regression equation: I (mA) = 0.013 [Glucose] (mM)–0.33 and a correlation coefficient (R^2^) of 0.99. The SPCE/CuS-2 sensor demonstrated a good analytical performance, with a low limit of detection (LoD) of 0.2 mM, a sensitivity of 0.013 mA/mM, and a wide dynamic linear range (DLR) from 0.2 to 16 mM. This confirmed that the developed sensor could potentially measure glucose levels in diabetic patients suffering from hypoglycemia (low blood sugar levels) and hyperglycemia (high blood sugar levels) (i.e., high blood sugar levels). To assess the performance of our proposed glucose sensor, we compared it to some previously reported CuS-nanoparticles-based glucose sensors, as shown in Table S1. The SPCE/CuS-2 sensor demonstrated equivalent sensitivity to the other sensors without the use of any binder materials or doping nanomaterials as support-catalysts to enhance the electrocatalytic activity of CuS. The overall performance of the SPCE/CuS-2 sensor was comparable to that of others, demonstrating a relatively high sensing ability as a result of the high electron transfer and improved electrode conductivity facilitated by the CuS-2 sensing film. The sensor showed good biocompatibility and structural simplicity, as it contains no complex mixture of catalysts. Therefore, one can alter conductivity and catalytic activity to acquire greater sensing capabilities by studying and perfecting the structural morphology, shape, and size of CuS nanoparticles. Additionally, most non-enzymatic sensors for glucose detection that were reported in the literature, including those for CuS and its composites, lack activity at physiological pH conditions (i.e., pH 7) and often exhibit strong catalytic ability for glucose at alkaline or basic pH conditions [21,22,23]. This hinders their ability to be used as enzyme substitutes for the practical analysis of glucose in biological fluids such as blood. Subsequently, the non-enzymatic nanomaterial-based sensors for glucose, as described in the recent literature, exhibit narrow dynamic linear ranges, which are often not suited for real analysis of glucose in actual blood samples [19,21,26].

### 3.8. Interference Studies of CuS-2-Modified Electrodes

**Figure 9:**
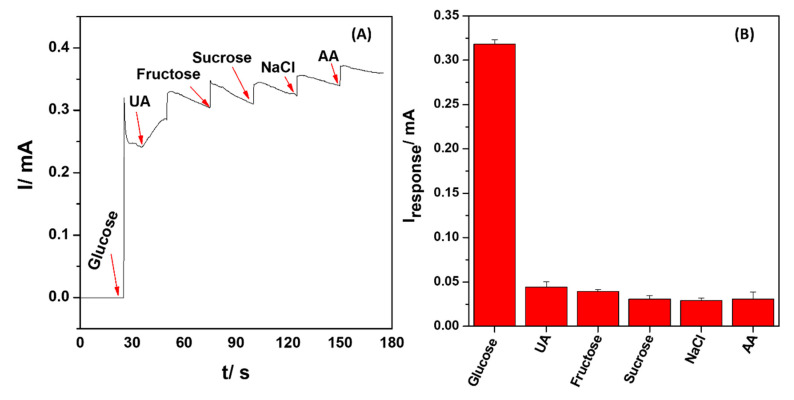
(**A**) Chronoamperometric responses of SPCE/CuS-2 in 3 mM glucose and 0.1 mM of the interference species; UA, fructose, sucrose, NaCl, and AA. (**B**) The plot of interfering current signal to the current response of glucose.

One of the most difficult challenges in non-enzymatic glucose detection is reducing the electrochemical response caused by other electroactive species commonly found in blood samples. These species include uric acid (UA), ascorbic acid (AA), and other carbohydrates compounds. The physiological concentration of glucose in the blood is about 3–8 mM, while that of AA and UA is about 0.1 mM. These interfering species can produce redox potentials close to that of glucose [70,71,72]; hence, the effect of interference on the sensing platform becomes a very crucial tool during the development of electrochemical sensors. The selectivity of the SPCE/CuS-2 sensor toward glucose was validated by using chronoamperometry by the successive addition of 0.1 mM of the interference species uric acid (UA), fructose, sucrose, NaCl and ascorbic acid (AA), in the presence of 3 mM glucose, as illustrated in Figure 9A, at the applied potential of 0.65 V. The SPCE/CuS-2 sensor showed the highest electrocatalytic response to glucose when compared to the other measured interference species, which had insignificant current responses of less than 0.05 mA. Figure 9B depicts the current responses for various interference species. The results demonstrated that the developed SPCE/CuS-2 sensor is highly selective for glucose, has good anti-interference capability, and could potentially be applied for practical detection of glucose in real samples.

### 3.9. Blood Sample Analysis

Diabetes can be effectively managed by measuring blood glucose concentrations, using either blood or serum samples. The CuS-2-modified electrode was used to detect glucose in blood samples by using the standard addition method or spiking without sample pretreatment to explore the potential application of the fabricated sensor in a real sample analysis [73,74]. Spiking, percentage recovery, and linearity of glucose experiments are all effective techniques for validating and evaluating a sensor’s accuracy. Spiking and recovery are used to determine whether glucose detection is affected by differences in the standard curve of pure glucose and blood sample matrix. Blood matrix can be either a pure (undiluted) biological sample or a blood mixture spiked with pure glucose solution. The amount of analyte in the spike causes the difference in analytical response between spiked and unspiked samples. This serves as a reference point for determining the analyte concentration in the original sample [73,74].

The concentration of glucose in whole blood was determined to be 4.7 mM by a commercial glucose meter. The recovery tests were performed by spiking 1–4 mM of pure glucose solution into the blood samples and measuring the current response at 0.65 V for five measurements for each sample. Table 2 shows the comparison results between the spiked values and the determined values. It can be seen that the recoveries for the determination of glucose are between 96.6% and 110%. However, the sample spiked with 0.5 mM of pure glucose gave inadequate results, and the concentration of glucose was found to be noticeably lower than the concentration of glucose added. This might due to the fact that the amount of glucose added was significantly small even that it could not increase the ion signal intensities for the analyte of interest—in this case, glucose. On the other hand, chloride ions, which are abundant in human blood, are considered to be the primary external poisoning species for noble-metal-based non-enzymatic glucose sensors, resulting in a rapid decrease in current response on the working electrode [75,76]. The obtained results revealed the potential applications of the SPCE/CuS-2 sensor for glucose sensing in blood samples.

## 4. Conclusions

Cu_x_S_y_ nanomaterials were created by using a simple wet chemical method. The method has the potential for scalability due to its simplicity and control over variable parameters. The synthesized CuS nanostructures showed improved electrocatalyic activity. The non-enzymatic sensor based on the CuS-2-nanomaterials-modified SPCE demonstrated good reproducibility, selectivity, and high electrocatalytic activity toward glucose attributed to the large nanoparticle size (25 ± 0.6 nm) of CuS-2. The SPCE/CuS-2 sensor demonstrated good catalytic performance with measurable parameters such as a low detection limit (LoD) of 0.2 mM and a wide dynamic linear range from 0.2 to 16 mM toward the detection of glucose, as well as a high potential for use in real-time glucose determination in whole blood. The findings from this study indicate that particle size has a significant impact on the material’s electrochemical performance. Larger nanoparticles are preferable since they promote conductivity and high electron transfer due to their large electrochemical surface coverage.

## Figures and Tables

**Table 1:** EIS data of the Bare SPCE, SPCE/CuS-1, and SPCE/CuS-2 in [Fe(CN)_6_]^3−^/[Fe(CN)_6_]^4−^.

Electrodes	EIS Parameters			
	R_s_ (Ω)	R_ct_ (Ω)	C_dl_ (µF)	CPE (µF)
Bare SPCE	642	201	8.62	114
SPCE/CuS-1	930	536	1.61	57
SPCE/CuS-2	486	93	1.56	590

**Table 2:** Determination and recovery of glucose in spiked blood samples, using SPCE/CuS-2 sensor.

Samples	Added Conc.	Found Conc.	RSD (%)	Recovery %
**1**	0.5 mM	−0.2	7.01	40
**2**	1 mM	1.1 mM	5.33	110
**3**	2 mM	2.1 mM	3.13	105
**4**	3 mM	2.9 mM	4.72	96.66
**5**	4 mM	4.0 mM	6.99	100

## Data Availability

Data can be available upon request from the authors.

## Supplementary Materials

The following supporting information can be downloaded at https://www.mdpi.com/article/10.3390/nano13030481/s1. Figure S1: TEM image of CuS-1 at 50 nm magnification scale. Figure S2: The SEM images of (a) SPCE/CuS-2 and (b) SPCE/CuS-1. Figure S3: Plot of anodic peak potential (Ep) versus log v for SPCE/CuS-2. Table S1: Comparison of the fabricated CuS glucose sensor with other reported non-enzymatic glucose sensors. References [77,78,79,80,81] are cited in the supplementary materials.
