# Supplementary material for: Synthesis, Characterization, and Electrochemical Evaluation of Copper Sulfide Nanoparticles and Their Application for Non-Enzymatic Glucose Detection in Blood Samples
Source: Nanomaterials (Basel). 2023 Jan 25;13(3):481. doi: 10.3390/nano13030481 (PMC9919628; doi:10.3390/nano13030481)
Supplement: Supplementary file 1 [file nanomaterials-13-00481-s001.zip › nanomaterials-1846421-supplementary.pdf]

Supplementary Information

Synthesis, Characterization, and Electrochemical Evaluation of Copper Sulfide Nanoparticles and Their Application for Non-Enzymatic Glucose Detection in Blood Samples

Phumlani Tetyana [1,2,], Ntsoaki Mphuthi [1,3] Abongile Nwabisa Jijana [1,*], Nosipho Moloto [2], Poslet Morgan Shumbula [4], Amanda Skepu [5], Lea Sibulelo Vilakazi [1] and Lucky Sikhwivhilu [1,6,*]

1. DSI/Mintek Nanotechnology Innovation Centre, Advanced Materials Division, Mintek, Private Bag X3015, Randburg 2125, South Africa
2. Department of Chemistry, University of Witwatersrand, Private Bag X3, Braamfontein 2050, South Africa
3. Department of Chemical Sciences, University of Johannesburg, Doornfontein 2028, South Africa
4. Department of Chemistry, University of Limpopo, Private Bag X1106, Sovenga 0727, South Africa
Next Generation Health, Division 1, CSIR, Meiring Naude Road, Brummeria, Pretoria 0001, South Africa
6. Department of Chemistry, Faculty of Science, Engineering and Agriculture, University of Venda, Private Bag X5050, Thohoyandou 0950, South Africa

* Correspondence: abongilej@mintek.co.za (A.N.J.); luckys@mintek.co.za (L.S.)

S1. Diffusion coefficient

Diffusion coefficient was calculate to determine the rate at which the electrons diffuse from the electrolyte solution to the electrode surface using Randles-Sevcik Equation (S1) [1]:

$$I_p = 2.69\, x\, 10^5 A n^{3/2} D^{1/2} C v^{1/2} \quad \text{(S1)}$$

where *I*p is the peak current, *n* is the number of electrons involved in the $[Fe(CN)_6]^{3-}$/$[Fe(CN)_6]^{4-}$ redox process, *A* is the area of the electrode (0.0707 cm^2), *D* is the diffusion coefficient, *C* the concentration of ferricyanide solution (1 mM) and *υ* is the scan rate (Vs^{-1}). This equation was derived for the case of the diffusion of redox species whose bulk concentration is not massively changed during the reaction time [1].

S2. Surface Coverage

$$\theta = \left|1 - \frac{R_{ct}^{bare}}{R_{ct}^{modified}}\right| \quad \text{(S2)}$$

The surface coverage [2] is given by *θ*, parameters; R_{ct}^{bare} and $R_{ct}^{modified}$ are the charge transfer resistances of the bare SPCE and the SPCE/CuS-1, SPCE/CuS-2 modified electrodes respectively.

S3. Exchange Current Density

The exchange current density parameters were evaluated through Butler-Volmer equations [3–5];

$$R_{ct} = \frac{RT}{nFi_o} \quad \text{(S3)}$$

where R_{ct} is the charge transfer resistance, *R* is the gas constant, *T* is the temperature in Kelvins, *n* is the number of electrons transferred, *F* is the Faradays constant and i_o is the exchange current density.

S4. Tafel Equation

The Tafel slope was calculated from Tafel plot of anodic peak potential (E_p) versus log v (Figure S3) using the Tafel equations (S4) [6,7]:

$$E_P = \left(\frac{b}{2}\right)\log v + k \tag{S4}$$

Or

$$E_p = k + \frac{2.303RT}{2(1-\ \alpha)n_\alpha F}\log v \tag{S5}$$

where b is the Tafel value k is a constant, α is the transfer coefficient, R is gas constant, T is temperature and F is faraday's constant. The Tafel slope values were calculated using equation S4.

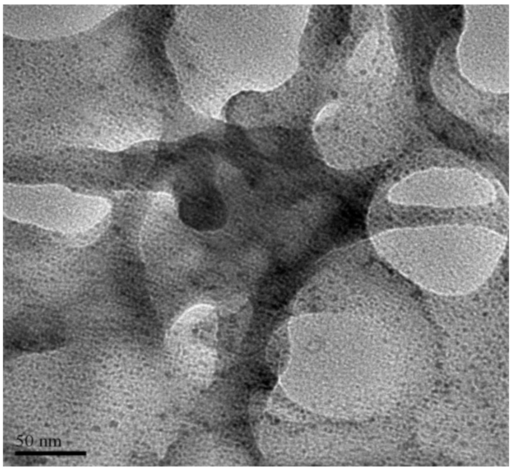

Figure S1. TEM image of CuS-1 at 50 nm magnification scale.

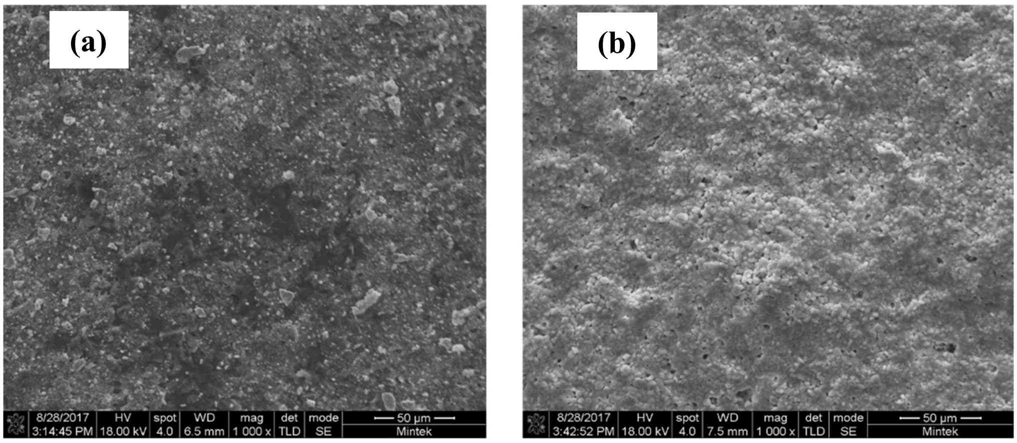

Figure S2. The SEM images of (a) SPCE/CuS-2 and (b) SPCE/CuS-1.

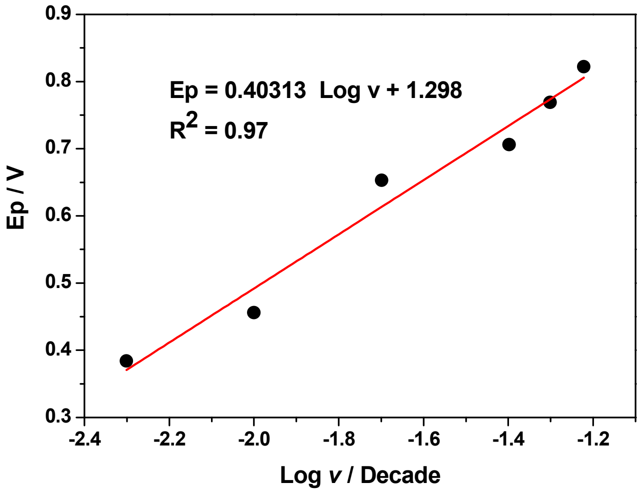

Figure S3. Plot of anodic peak potential (Ep) versus log v for SPCE/CuS-2.

Table S1. Comparison of the fabricated CuS glucose sensor with other reported non-enzymatic glucose sensors.

Sensor	Sensitivity (µA/mM)	LOD (µM)	Linear range	Detection potential (V)	Electrolyte	References
**CuS/CS/GCE**	5.86	0.3	1µm–0.1mM	0.46	PBS pH 7.2	[8]
**CuS/NSC/GCE**	13620	2.72	160 mM–11.76 mM,	0.5	0.1 M NaOH	[9]
**CuS MF/GCE**	1007	2	0.02–5.4 mM	0.5	0.1 M NaOH	[10]
**$CuS/Cu_2S/GCE$**	321.3	1.1	3.0–1100.0 µM	0.6	0.1M NaOH	[11]
**$CuS/Cu_2O/CuO/Cu$**	4262	0.89	0.002–4.096 mM	0.6	0.1 M NaOH	[12]
**CuS/RGO/CuS/Cu**	1054	0.5	1–665µM	0.65	0.1 M NaOH	[13]
**V-CuS/GCE**	1800	0.4	2 µM–5 mM	0.5	0.1 M NaOH	[14]
**h-CuS NCs/Nafion/GCE**	7180	-	0.1–10 mM	0.5	0.1M NaOH	[15]
**Pt/replaced/Cu/SPE**	9.62	0.385 mM	1–11 mM	0.4	PBS pH 7.2	[16]
**rGO/CuS/GCE**	76.27	-	3.88–20.17 mM	0.48	0.1 M NaOH	[17]
**CuS-HCM/GCE**	64.97	3.78	0–12 mM	0.7	0.1 M NaOH	[18]
**CuS/SPE**	13	0.2 mM	1–16 mM	0.65	PBS pH 7.4	This work

Reference

1. Bian, C.-L.; Zeng, Q.-X.; Yang, L.-J.; Xiong, H.-Y.; Zhang, X.-H.; Wang, S.-F. Voltammetric studies of the interaction of rutin with DNA and its analytical applications on the MWNTs–COOH/Fe_3O_4 modified electrode. *Sens. Actuators B Chem.* **2011**, *156*, 615–620.
2. Ndangili, P.M.; Jijana, A.N.; Olowu, R.A.; Mailu, S.N.; Ngece, F.R.; Williams, A.; Waryo, T.T.; Baker, P.; GL; Iwuoha, E.I. Impedimetric Response of a Label-Free Genosensor Prepared on a 3-Mercaptopropionic Acid Capped Gallium Selenide NanocrystalModified Gold Electrode. *Sch. Pure Appl. Sci.* **2011**, *6*, 1438–1453.
3. Sacco, A. Electrochemical impedance spectroscopy: Fundamentals and application in dye-sensitized solar cells. *Renew. Sustain. Energy Rev.* **2017**, *79*, 814–829.
4. Survila, A.; Mockus, Z.; Kanapeckaitė, S.; Grigucevičienė, A.; Stalnionis, G. EIS characterization of Sn|Sn(II), gluconic acid system. *Electrochim. Acta* **2012**, *85*, 594–599.
5. Guiñón-Pina, V.; Igual-Muñoz, A.; García-Antón, J. Influence of temperature and applied potential on the electrochemical behaviour of nickel in LiBr solutions by means of electrochemical impedance spectroscopy. *Corros. Sci.* **2009**, *51*, 2406–2415.
6. Adekunle, A.S.; Lebogang, S.; Gwala, P.L.; Tsele, T.P.; Olasunkanmi, L.O.; Esther, F.O.; Boikanyo, D.; Mphuthi, N.; Oyekunle, J.A.O.; Ogunfowokan, A.O.; et al. Electrochemical response of nitrite and nitric oxide on graphene oxide nanoparticles doped with Prussian blue (PB) and Fe_2O_3 nanoparticles. *RSC Adv.* **2015**, *5*, 27759–27774

7. Soderberg, J.N.; Co, A.C.; Sirk, A.H.C.; Birss, V.I. Impact of Porous Electrode Properties on the Electrochemical Transfer Coefficient. *J. Phys. Chem. B* **2006**, *110*, 10401–10410.
8. Yang, Y.J.; Zi, J.; Li, W. Enzyme-free sensing of hydrogen peroxide and glucose at a CuS nanoflowers modified glassy carbon electrode. *Electrochim Acta*. **2014**, *115*, 126–130
9. Sharma, K.P.; Shin, M.; Awasthi, G.P.; Poudel, M.B.; Kim, H.J.; Yu, C. Chitosan polymer matrix-derived nanocomposite (CuS/NSC) for non-enzymatic electrochemical glucose sensor. *Int. J. Biol. Macromol*. **2022**, *206*, 708–717.
10. Radhakrishnan, S.; Kim, H.-Y.; Kim, B.-S. A novel CuS microflower superstructure based sensitive and selective nonenzymatic glucose detection. *Sens. Actuators B Chem*. **2016**, *233*, 93–99.
11. Huang, W.; Liu, F.; Huang, Y.; Yang, W.; Zhong, H.; Peng, J. Facile One-pot Synthesis of Hollow-structured CuS/Cu_2S Hybrid for Enhanced Electrochemical Determination of Glucose. *Electrochemistry* **2021**, *89*, 340–347.
12. Wei, C.; Zou, X.; Liu, Q.; Li, S.; Kang, C.; Xiang, W. A highly sensitive non-enzymatic glucose sensor based on CuS nanosheets modified Cu_2O/CuO nanowire arrays. *Electrochim. Acta* **2020**, *334*, 135630.
13. Zhao, C.; Wu, X.; Zhang, X.; Li, P.; Qian, X. Facile synthesis of layered CuS/RGO/CuS nanocomposite on Cu foam for ultrasensitive nonenzymatic detection of glucose. *J Electroanal Chem*. **2017**, *785*, 172–179.
14. Gao, P.; Zhang, Y.; Abedi, H. Hierarchical CuS doped with vanadium nanosheets with micro skein overall morphology as a high performance amperometric glucose sensor. *Surf. Interfaces* **2020**, *21*, 100756.
15. Zhu, J.; Peng, X.; Nie, W.; Wang, Y.; Gao, J.; Wen, W.; Selvaraj, J.N.; Zhang, X.; Wang, S. Hollow copper sulfide nanocubes as multifunctional nanozymes for colorimetric detection of dopamine and electrochemical detection of glucose. *Biosens. Bioelectron*. **2019**, *141*, 111450.
16. Hu, Y.; Niu, X.; Zhao, H.; Tang, J.; Lan, M. Enzyme-Free Amperometric Detection of Glucose on Platinum-Replaced Porous Copper Frameworks. *Electrochim Acta.* **2015**, *165*, 383–389.
17. Karikalan, N.; Karthik, R.; Chen, S.-M.; Karuppiah, C. Elangovan A. Sonochemical Synthesis of Sulfur Doped Reduced Graphene Oxide Supported CuS Nanoparticles for the Non-Enzymatic Glucose Sensor Applications. *Sci Rep*. **2017**, *7*, 2494.
18. Liu, X.; Ai, L.; Jiang, J. Interconnected porous hollow CuS microspheres derived from metal-organic frameworks for efficient adsorption and electrochemical biosensing. *Powder Technol.* **2015**, *283*, 539–548.
